# Identification of heterophyllin B accumulation associated genes via WGCNA in *Pseudostellaria heterophylla*

**DOI:** 10.3389/fpls.2025.1691269

**Published:** 2025-12-09

**Authors:** Li-Yi Xu, Shuai Wang, Qing-Lin Xu, Hong-Song Bu, Ying-Ying Wu, Jing-Jing Su, Zu-Yun Ye

**Affiliations:** 1 College of Biological Science and Engineering, Ningde Normal University, Ningde, China; 2 The Engineering Technology Research Center of Characteristic Medicinal Plants of Fujian, Ningde Normal University, Ningde, China

**Keywords:** medicinal plants, heterophyllin B, gene identification, weighted gene co-expression network analysis, *Pseudostellaria heterophylla*

## Abstract

*Pseudostellaria heterophylla* (Miq.) Pax (*P. heterophylla*) is a perennial medicinal herb in which heterophyllin B (HB) serves as one of the primary bioactive compounds. Identifying genes associated with HB accumulation is crucial for breeding high-HB cultivars. In this study, we performed HPLC quantification and high-throughput RNA sequencing on three *P. heterophylla* accessions with differential HB content. Weighted gene co-expression network analysis (WGCNA) of the assembled transcriptome identified HB accumulation-associated modules, followed by qRT-PCR validation of candidate genes. HPLC quantification revealed significant variation in HB content among three samples (59.48 - 369.63 μg/g), with the sample ZS2 (a provincial-certified cultivars) identified as an HB-deficient genotype (t-test, *p* < 0.01 for all pairwise comparisons). *De novo* transcriptome assembly using Trinity generated a reference sequence comprising 114,625 transcripts, achieving 89.96 - 92.37% clean read mapping rates across samples. WGCNA clustered expressed genes into 55 modules, among which the yellow module (3,240 genes) showed the strongest positive correlation with HB accumulation. Gene Significance (GS) and Module Membership (MM) evaluation further identified 278 high-confidence candidate genes within this module. qRT-PCR validation using 180-day tissue-cultured samples confirmed one gene (*g3166_i1*) exhibiting perfect positive correlation with HB variation (kendall’s τ = 1). This study delineates transcriptomic signatures underlying HB divergence in *P. heterophylla* and provides actionable genetic targets for molecular breeding of high-HB cultivars.

## 1 Introduction

*Pseudostellaria heterophylla* (Miq.) Pax ex Pax et Hoffm. (*P. heterophylla*) is a perennial herb with well-known medicinal value (Li et al., 2022; Yang et al., 2003), primarily cultivated for its dried tuberous roots in China, Korea, and neighboring regions (Choi and Pak, 2000; Liu et al., 2017). In China, *P. heterophylla* is predominantly cultivated in Fujian, Guizhou, Jiangsu, and Shandong provinces (Kang et al., 2016). With over a century of clinical application history (Hu et al., 2019), it has been traditionally used to treat fatigue, spleen deficiency, anorexia, post-illness debility, and dry cough induced by lung dryness (Xiao et al., 2022; Zhang et al., 2024). Its mild pharmacological properties make it particularly suitable for pediatric use, earning it the vernacular name “Hai Er Shen” (phonetically akin to the Chinese word for “children”) in Chinese. Heterophyllin B (HB) is one of the trace active components in *P. heterophylla* as a medicinal material (Wang et al., 2013). According to the 2022 standards (Standard number: YBZ-PFKL-2022046) issued by the Chinese Pharmacopoeia Commission, each gram of *P. heterophylla* formula granules should contain 0.3 mg to 0.65 mg of HB. Currently, due to long-term reliance on vegetative propagation (Gu et al., 2025), *P. heterophylla* has limited cultivated varieties (Xu et al., 2023), and most exhibit HB content only marginally above the standard threshold. Therefore, there is an urgent need to breed *P. heterophylla* cultivars with high HB content to provide reliable plant materials for more effective exploitation and utilization of this medicinal resource.

Plant cyclic peptides (CPs) represent a major class of small-molecule metabolites, typically formed through the cyclization of 2–37 proteinogenic and non-proteinogenic amino acids (usually L-amino acids) via peptide bonds (Burman et al., 2010; Kaleem et al., 2012). These bioactive peptides are widely distributed in various tissues of higher plants, including stem bark, leaves, seeds, and roots (Condie et al., 2011; Xu et al., 2012). Cyclic peptides isolated from *P. heterophylla* exhibit relatively simple structures compared to those from other plant species, primarily categorized into two structural types: heterophyllin-type peptides and pseudostellarin-type peptides (Yang et al., 2025; Daly and Wilson, 2021). Among them, HB, a cyclic octapeptide, consists of eight L-amino acids linked by peptide bonds to form a single cyclic structure (Jia et al., 2006). In China, HB was first adopted as one of the quality control markers for *P. heterophylla* in 2010 (Zhao et al., 2015). Current research has demonstrated that HB exhibits significant pharmacological efficacy, particularly in anti-inflammatory (Yang et al., 2018), blood glucose regulation (Liao and Tzen, 2022), and memory enhancement applications (Yang et al., 2021; Deng et al., 2022). However, the metabolic pathways underlying HB biosynthesis remain poorly characterized. Identifying the genes involved in HB production is therefore critical for understanding the molecular mechanisms governing HB accumulation in *P. heterophylla*. Such research would enable efficient development of high-HB cultivars through molecular breeding strategies.

In molecular breeding research, correlating phenotypic data with transcriptomic data enables rapid identification of candidate genes associated with target traits. Weighted Gene Co-expression Network Analysis (WGCNA) is a genetic methodology that analyzes gene-gene correlations using large-scale gene expression profiles (Ruan et al., 2010; Fuller et al., 2011). This approach clusters tens of thousands of genes from transcriptomic datasets into dozens of gene modules and associates these modules with specific traits or phenotypes, thereby reducing the complexity of functional gene selection (Stuart et al., 2003). Particularly suitable for investigating relationships between functional modules and phenotypic traits across multiple samples, WGCNA has been successfully applied in various plant species including rice (Wang et al., 2022), maize (Ma et al., 2021), and tea plant (Xu et al., 2025) as an effective method for detecting co-expression modules and genes related to target phenotypes. In this study, we therefore adopted this WGCNA-based strategy to systematically identify genes associated with HB accumulation.

Herein we cultivated and examined three *P. heterophylla* samples exhibiting differential HB accumulation, and generated their transcriptome profiles using next-generation sequencing. This study aims to (1) quantify inter-sample HB variation in 180-day-old plants through HPLC analysis; (2) construct a WGCNA from RNA-seq data to identify HB-associated expression modules; (3) identify key genes within the modules that are associated with target traits, providing a reliable basis for future breeding of high-HB *P. heterophylla* varieties.

## 2 Materials and methods

### 2.1 Plant materials

Three types of *P. heterophylla* (‘ZS1’, ‘ZS2’, and ‘ZS5’) were selected for this study. Samples ‘ZS1’ and ‘ZS2’ were provincially certified cultivars, whereas ‘ZS5’ represented a farmer-preferred landrace.

For long-term preservation, the plant materials were maintained in two forms: (1) asexually propagated clones housed at the Engineering Technology Research Center for Characteristic Medicinal Plants, Fujian, Ningde Normal University, and (2) field-grown plants cultivated at the *P. heterophylla* Breeding Base (Yingshan Township, Zherong County, Fujian, China). For field-grown plants, newly generated tuberous root tissues were harvested at 180 days post-transplantation for transcriptome sequencing. For tissue-cultured samples, we collected mature micro-tuberous root after 180 days of primary culture for qPCR validation experiments.

### 2.2 HB extraction, HPLC analysis and calibration curve construction

For accurate HB quantification, HPLC analysis was performed. The tuberous roots were ground into powder, and 2.0 g of powder was extracted with 50.0 mL methanol by ultrasonication (250 W, 30 kHz) for 45 min. After cooling and filtration, 25.0 mL filtrate was transferred to a round-bottom flask and concentrated to dryness using a rotary evaporator. The residue was dissolved in methanol and diluted to 10.0 mL as the test solution.

Chromatographic separation was performed on an Agilent 1200 system (Agilent, USA) equipped with a C18 column (4.6 × 250 mm, 5 μm). The mobile phase consisted of acetonitrile (A) and aqueous phosphoric acid (0.2%, B) at a flow rate of 1.0 mL/min (isocratic elution). Detection was at 203 nm with the column maintained at 30°C and injection volume of 20.0 μL.

An HB reference standard (100.0 mg) was dissolved in methanol and diluted to 1000.0 mL to prepare a stock solution (100 μg/mL). Standard solutions (10-60 μg/mL, at 10 μg/mL intervals) were prepared by serial dilution. After filtration (0.22 μm), 20.0 μL of each solution was injected, with methanol as blank. The calibration curve was generated by plotting peak area (y-axis) versus concentration (x-axis), and the regression equation was calculated.

### 2.3 RNA extraction, library preparation, and high-throughput sequencing

Total RNA was extracted from *P. heterophylla* tuberous root tissues using a column-based plant RNA extraction kit (Sangon Biotech, Shanghai, China). RNA purity and concentration were determined using a Nanodrop-2000 spectrophotometer (Thermo Fisher Scientific, USA), and the samples were stored at -80°C until further analysis. The cDNA library construction and high-throughput sequencing were performed by Tsingke Biotechnology (Beijing, China) on an MGI sequencing platform.

### 2.4 Transcriptome data processing, construction of WGCNA, candidate gene identification

Raw sequencing reads were quality-assessed using FastQC, followed by filtering to remove low-quality sequences (Q-score < 30). Due to the absence of a reference genome for *P. heterophylla*, clean reads were *de novo* assembled into reference transcripts using Trinity. Processed reads were then aligned to the assembly using HISAT2 (Guo et al., 2022), and transcript abundances were quantified with StringTie (Pertea et al., 2015). Based on TMM-normalized expression values, principal component analysis (PCA) was conducted to evaluate inter-sample variation and replicate consistency.

Weighted gene co-expression network analysis was performed using R v4.4.1 (wgcna package) (Langfelder and Horvath, 2008). A soft-thresholding power (β, with a scale-free topology fit index > 0.8) was applied to construct unsigned networks. Genes were clustered into modules (mergeCutHeight = 0.25, minModuleSize = 30). To evaluate co-expression relationships between modules and HB content, an eigengene adjacency matrix was calculated based on their correlation coefficients. Heatmap visualization was performed to assess module-trait associations, enabling identification of HB-related key candidate modules for subsequent critical gene selection.

Key candidate genes were selected from target modules using stringent criteria: gene significance for HB content (|GS| > 0.9), module membership (MM > 0.9), *p* value of GS < 0.00001.

### 2.5 Validation and quantitative real-time PCR of candidate genes

We verified WGCNA-identified candidate genes by measuring HB content and performing qRT-PCR analysis in tuberous root tissues of three field-grown samples (‘ZS1’, ‘ZS2’, and ‘ZS5’). Total RNA was extracted from tuberous roots using the Column-based Plant RNA Extraction Kit (Sangon Biotech, China). The extracted RNA was then treated with MightyScript plus Master Mix (Sangon Biotech, China) for gDNA removal and cDNA synthesis. Gene-specific primers were designed using Primer-BLAST (**Table 1**). Quantitative real-time PCR (qPCR) was carried out on a QuantStudio 3 system (Thermo Fisher Scientific, USA) with 2× HyperScript SYBR Green Master Mix (Sangon Biotech, China) under the following conditions: (1) initial denaturation at 95°C for 15 sec; (2) 40 cycles of 95°C for 15 sec and 60°C for 30 sec; (3) melt curve analysis. *P. heterophylla* Actin1 (*PhACTIN1*, **Table 1**) served as the internal control. Gene expression was quantified via the 2^−ΔΔCt^ method (Livak and Schmittgen, 2001) with three biological replicates (each with three technical replicates). Statistical analysis was performed using Student’s t-test (p<0.05) in R v4.4.1 (stats package), with significance visualized via ggplot2 package (*p<0.05, **p<0.01) (Wickham, 2011).

**Table 1:** Summary of primers used in qRT-PCR.

Gene	Upstream primer	Downstream primer
*PhACTIN1*	CTGTATTTACGCTCAGGTGG	CATTGTGCTCAGTGGTG
*g3166_i1*	GGGGATGGAGTTGGTTGGAG	GAGTTGATGGGGACTGACGG
*g14986_i0*	GCCCCCAGTGTGGATTTGTA	TAACCTGCTCACCGTCACAC
*g688_i9*	GTCGAAAGGGTTTTCACCGC	CCAACTGACGACCCAATCCA

## 3 Results

### 3.1 Quantification of HB in *P. heterophylla* tuberous roots

**Figure 1:**
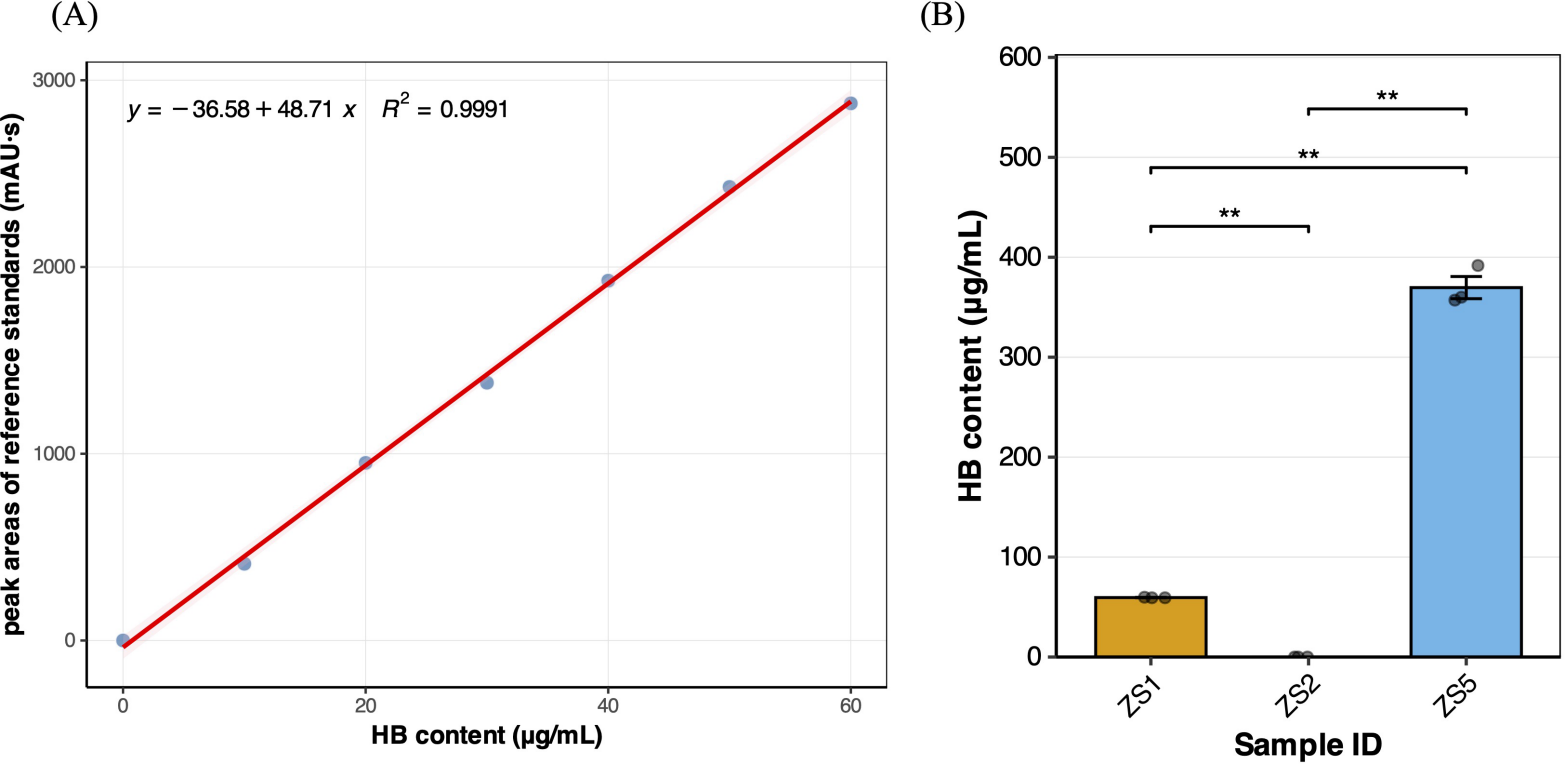
**(A)** Calibration curve for Heterophyllin B. **(B)** Heterophyllin B content variation among three field-grown samples, significant symbol “**” means the *p_value* < 0.01.

To assess whether significant differences in HB content existed among field-grown tuberous roots of different samples, we performed HPLC analysis on tree field-grown samples. This comparison was particularly relevant that the roots of *P. heterophylla* serve as the primary medicinal organs. A calibration curve (**Figure 1A**) was first established using HB reference standard solutions (10–60 μg/mL), which showed excellent linearity (R^2^ = 0.9991), demonstrating high reliability for subsequent sample quantification. HPLC analysis revealed that only ZS5 contained HB at 369.63 ± 11.09 μg/g (ZS1 only 59.48 ± 0.18 μg/g), meeting the Chinese pharmacopoeial standard (YBZ-PFKL-2022046) for drug ‘Taizishen Peifangkeli’ (> 300 μg/g). Notably, no detectable HB was observed in ZS2, suggesting it to be an HB-deficient genotype (**Figure 1B**). After the pairwise t-tests for significant differences, it was found that there were significant differences in the HB content among the samples (*p* < 0.01), which making them ideal materials for transcriptome-based identification of HB-related genes.

### 3.2 Transcriptome data processing

Following high-throughput sequencing, the samples exhibited original read counts ranged from 43,501,766 to 44,080,074 reads, with GC content ranging from 42.93% to 43.38% (**Table 2**). Quality assessment using FastQC program revealed that over 98% of reads achieved Q30 and over 99% reached Q20 quality scores. Given the absence of a reference genome for *P. heterophylla* or any closely related species in the same genus, we performed no-reference genome assembly using Trinity program. The assembled sequences (comprising 114,625 transcripts, 85,592 unigenes, and 38,288 coding genes) as reference sequences for subsequent read mapping and quantification across all samples (**Table 3**). To evaluate the assembly quality of the reference sequences, we aligned chloroplast genome-encoded genes from *P. heterophylla* (NCBI accession number: OQ405025.1) to the assembled transcripts using NCBI BLAST+ program (-evalue 1e-10 -word_size 30). The BLASTN alignment (**Supplementary Table S1**) detected 73 out of 77 expected chloroplast genes (94.81%) in the reference transcripts, confirming high assembly integrity.

**Table 2:** The reads information in the transcriptome for each sample after high-throughput sequencing.

Sample	Total reads	Base numble	GC content	>Q20%	>Q30%
ZS1-R-1	43,920,070	6,574,122,102	43.23%	99.65%	98.62%
ZS1-R-2	43,501,766	6,507,043,776	43.38%	99.62%	98.51%
ZS1-R-3	43,937,250	6,580,032,198	42.96%	99.63%	98.53%
ZS2-R-1	44,080,074	6,598,385,696	43.15%	99.64%	98.55%
ZS2-R-2	43,639,534	6,533,360,860	43.20%	99.62%	98.52%
ZS2-R-3	43,520,062	6,513,427,716	42.93%	99.60%	98.43%
ZS5-R-1	43,555,968	6,519,419,522	43.07%	99.62%	98.50%
ZS5-R-2	43,920,070	6,560,961,690	43.22%	99.66%	98.69%
ZS5-R-3	43,560,064	6,518,350,588	43.17%	99.58%	98.35%

**Table 3:** The sequences length distribution of the assembled reference sequences.

Sequence type	200–500 bp	500–1000 bp	1000–2000 bp	> 2000 bp	Total
Transcripts	45,229	19,301	23,308	26,787	114,625
Unigenes	45,223	16,470	10,989	12,910	85,592
Coden genes	186	3,033	13,902	21,167	38,288

Using HISAT2 program, we aligned clean reads from each sample to the reference sequences. The results showed mapping rates of 89.96-92.37% across all samples (**Table 4**). Additionally, unique alignments accounted for 34.77-42.19% of total mapped reads.

**Table 4:** Alignment statistics of clean reads to reference sequences across samples.

Sample ID	Total reads	Unmapped reads (%)	Mapped reads (%)	Secondary alignments (%)	Unique alignments (%)
ZS1_R_1	43,920,070	4,299,073(9.79)	39,620,997(90.21)	22,578,213(51.41)	17,042,784(38.8)
ZS1_R_2	43,501,766	4,366,888(10.04)	39,134,878(89.96)	22,132,649(50.88)	17,002,229(39.08)
ZS1_R_3	43,937,250	3,512,531(7.99)	40,424,719(92.01)	22,058,159(50.2)	18,366,560(41.8)
ZS2_R_1	44,080,074	3,362,296(7.63)	40,717,778(92.37)	25,390,659(57.6)	15,327,119(34.77)
ZS2_R_2	43,639,534	4,005,142(9.18)	39,634,392(90.82)	21,445,755(49.14)	18,188,637(41.68)
ZS2_R_3	43,520,062	4,046,556(9.3)	39,473,506(90.7)	21,113,433(48.51)	18,360,073(42.19)
ZS5_R_1	43,555,968	3,946,983(9.06)	39,608,985(90.94)	22,460,849(51.57)	17,148,136(39.37)
ZS5_R_2	43,920,070	4,320,205(9.84)	39,599,865(90.16)	23,666,643(53.89)	15,933,222(36.28)
ZS5_R_3	43,560,064	4,307,122(9.89)	39,252,942(90.11)	21,863,847(50.19)	17,389,095(39.92)

**Figure 2:**
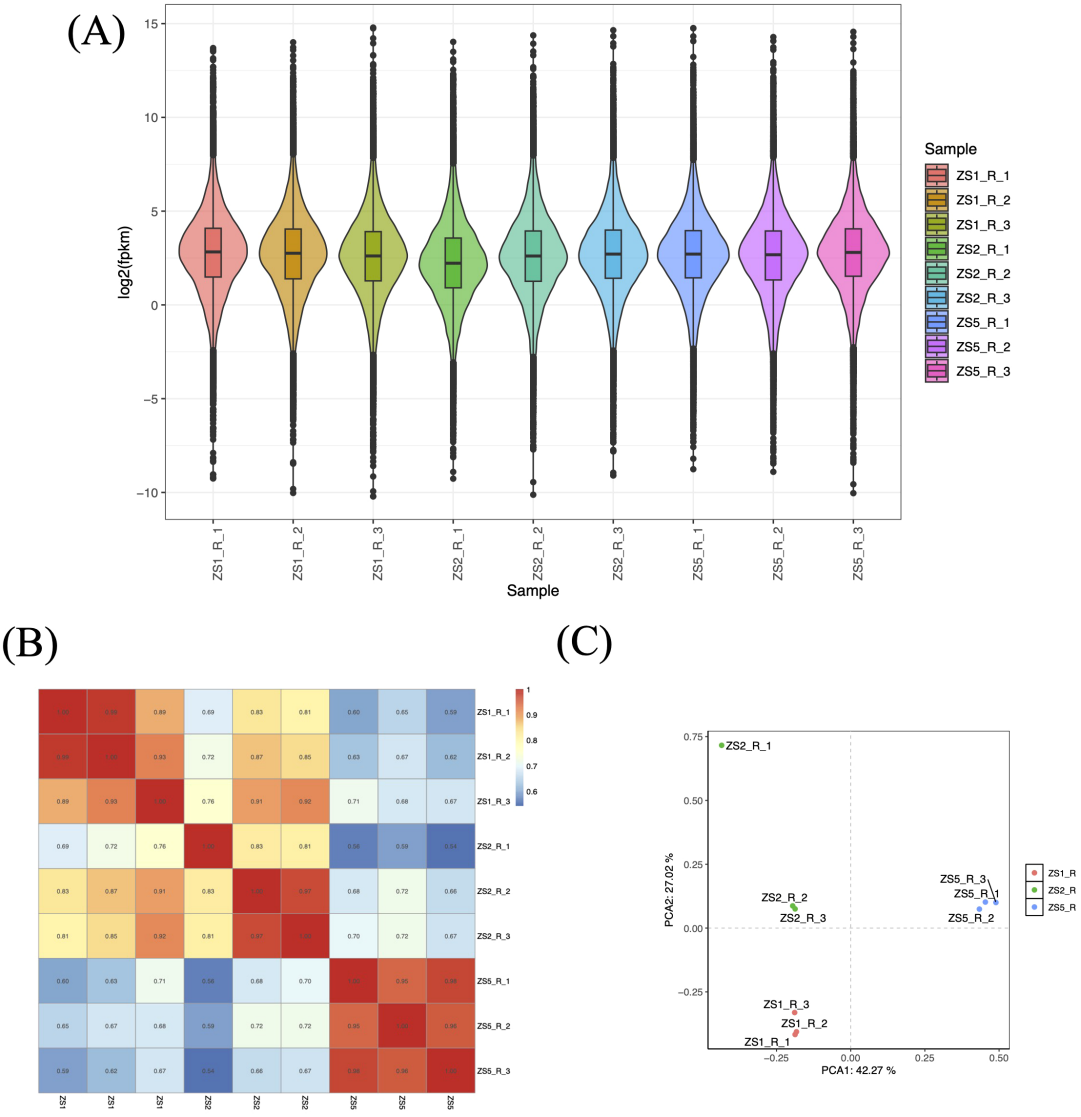
Analysis of gene expression patterns based on FPKM values. **(A)** Boxplot distribution of FPKM values across samples. Biological replicates are labeled as R_1 to R_3 following sample IDs. **(B)** Heatmap of correlation coefficients for inter-sample gene expression. The color gradient (blue to red) represents r values ranging from 0.6 to 1.0. **(C)** Principal component analysis (PCA) for inter-sample gene expression. Red, green, and blue points denote triplicate biological replicates of ZS1, ZS2, and ZS5, respectively.

Using StringTie program, we quantified gene expression abundance (Fragments Per Kilobase of transcript per Million mapped reads, FPKM) for all samples aligned to the reference sequences. All samples exhibited normally distributed FPKM values (**Figure 2A**). To assess inter-sample similarity and intra-sample reproducibility, we performed correlation analysis and principal component analysis (PCA), respectively. The correlation analysis revealed high reproducibility among biological replicates (r > 0.9), except for ZS2_R_1 (**Figure 2B**). Significant inter-sample variation was observed, with ZS5 showing distinct expression patterns. Notably, ZS2 replicates (ZS2_R_2 and ZS2_R_3) maintained moderate correlation with ZS1 (r > 0.8). PCA results corroborated these findings (**Figure 2C**), clearly separating the three sample groups into distinct clusters. However, ZS2_R_1 deviated substantially from its own replicates, indicating poor reproducibility. Based on these results, ZS2_R_1 was excluded from WGCNA analyses due to inconsistent reproducibility.

### 3.3 SSRs and SNPs discovery in *P. heterophylla* reference sequences

**Figure 3:**
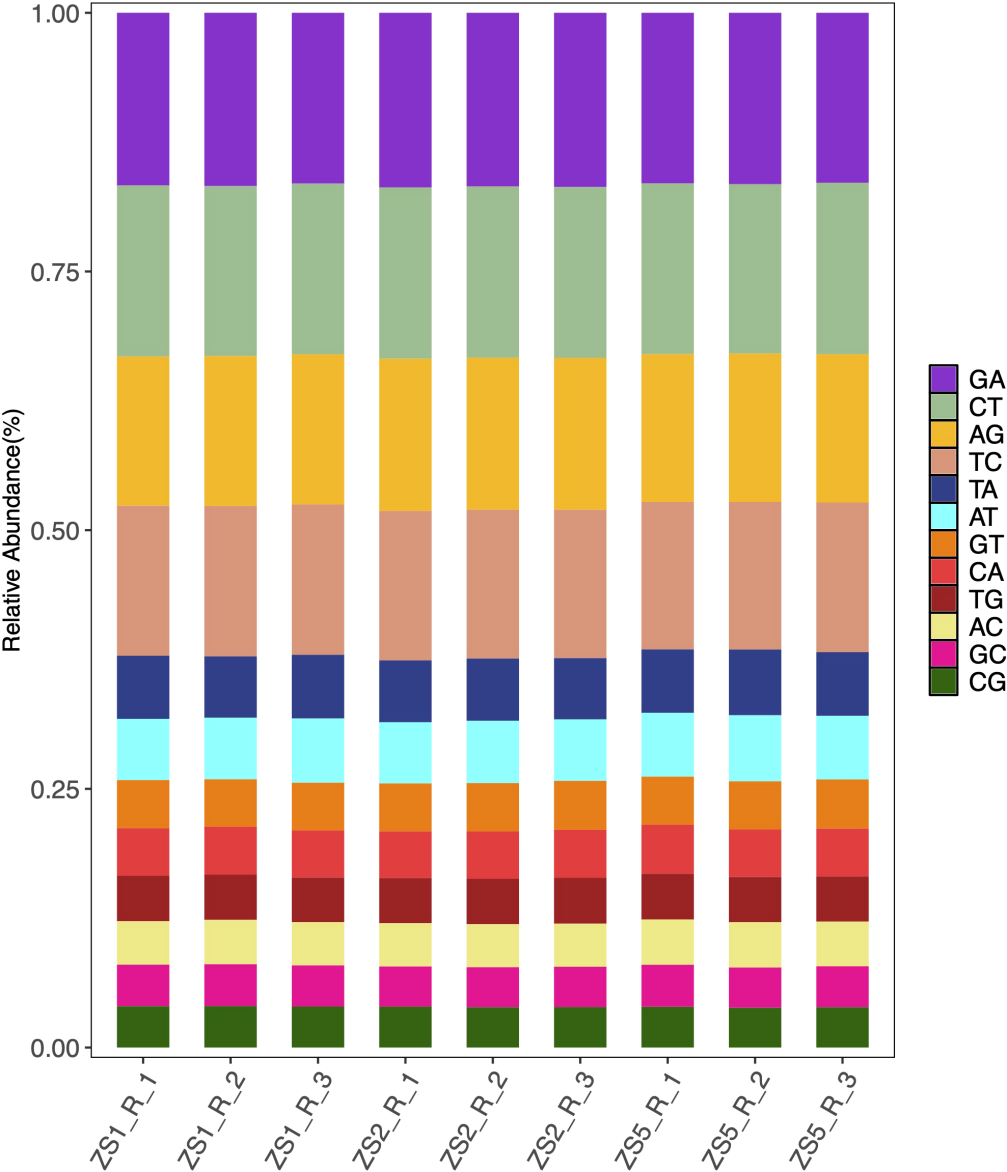
Distribution characteristics of SNP types across samples.

To facilitate genetic map construction and genome assembly for *P. heterophylla*, we identified SSR and SNP markers from the transcriptome sequences. A total of 2,330 SSR loci were detected within 2,053 coding genes (**Supplementary Table S2**), representing a 5.36% EST-SSR frequency among the 38,288 annotated coding genes. The SSR analysis revealed 1,890 loci (81.12%) contained dinucleotide or higher-order repeats, and among them, trinucleotide repeats predominated (1,222 loci, 52.45%). Alignment of all samples to the reference sequences revealed abundant SNP variants (from 76,301 to 89,726, **Supplementary Table S3**), with C/T (16.37 – 16.58%) and G/A (16.42 – 16.87%) transitions representing the most prevalent substitution types (**Figure 3**).

### 3.4 Construction of weighted gene co-expression networks and identifying modules associated with HB content

**Figure 4:**
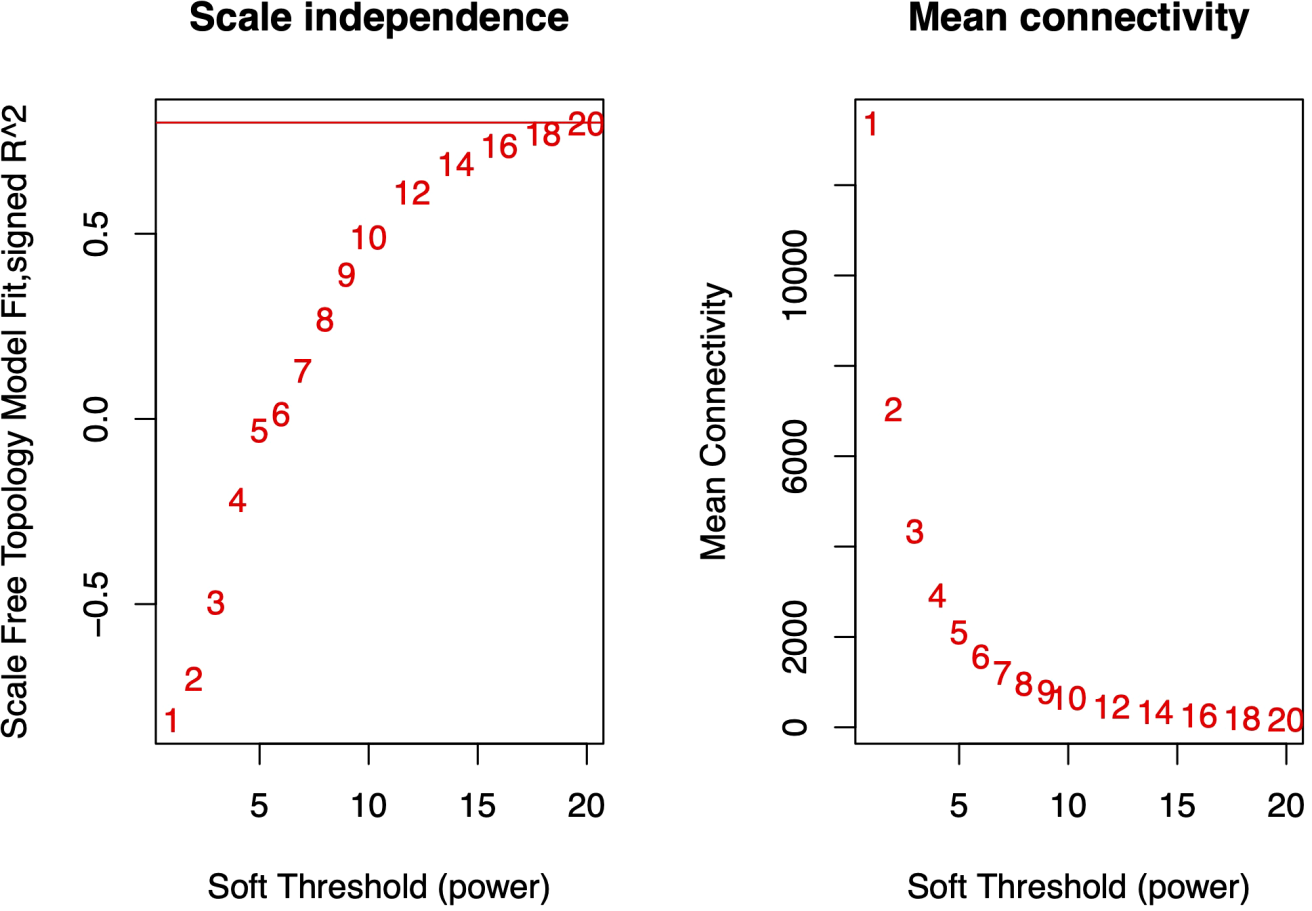
Scale-free topology fit index under varying soft-thresholding powers. The red horizontal line indicates the threshold of 0.8 for scale-free topology. At a soft-thresholding power (β) of 20, the fit index (R²) reached 0.8, suggesting the network approximates scale-free topology.

**Figure 5:**
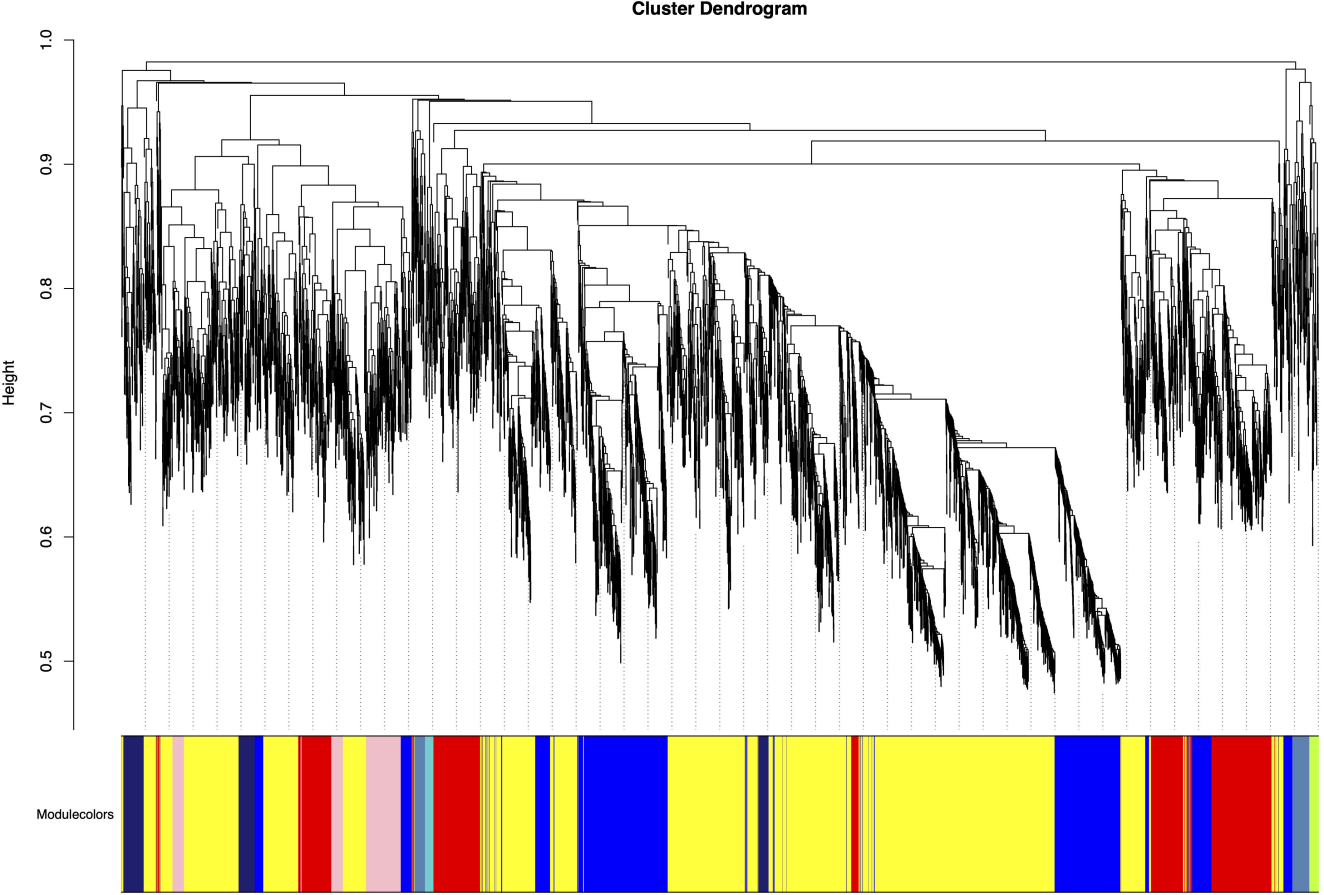
Gene modules identified by weighted gene co-expression network analysis (WGCNA). Modules were merged at a cut height of 0.25. Each colored row represents a distinct module, with color-coding indicating groups of highly interconnected genes. A total of 55co-expression modules was identified.

Using the gene expression abundance data (FPKM values) from Section 3.2, we constructed an expression matrix to calculate the scale-free topology fit index, which guided the selection of an appropriate soft-thresholding power. The analysis revealed that the scale-free topology fit index first exceeded 0.8 at a soft-thresholding power of 20 (**Figure 4**), which was subsequently adopted for network construction.The WGCNA network was built with the following parameters: minimum module size was 30 genes, module detection sensitivity with deepSplit was 2, module merging cut height was 0.25. This process ultimately generated a co-expression network comprising 55 distinct modules (**Figure 5**).

**Figure 6:**
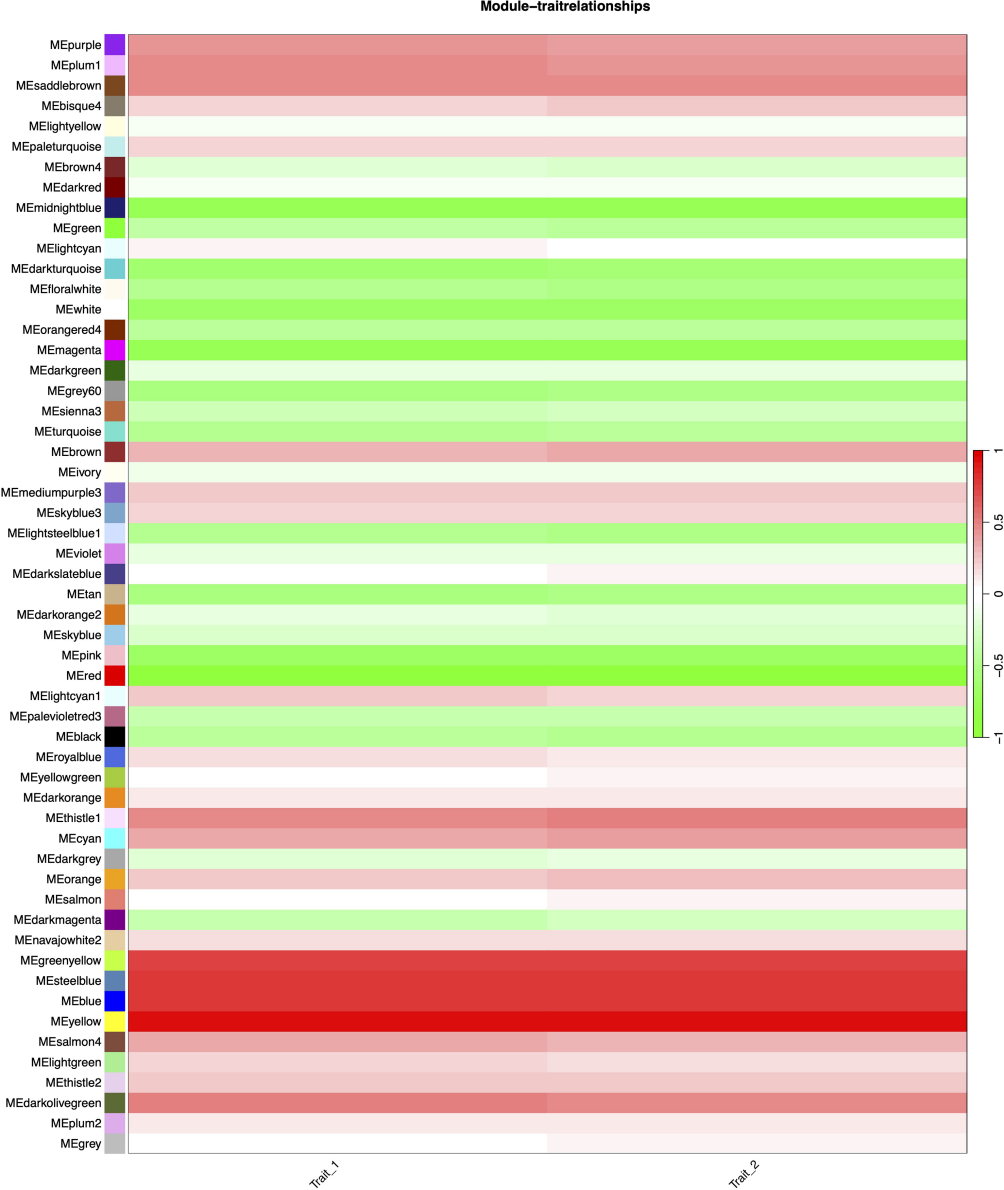
Heatmap of module-trait correlations in WGCNA analysis. Rows represent gene modules (labeled left of each cell) and columns represent traits. The color legend (right) indicates correlation strength: red for positive (max r = 1.0), green for negative (min r = -1.0), with gradient intensity reflecting magnitude. Trait_1: HB content in tissue-cultured plants; Trait_2: HB content in field-grown plants.

**Figure 7:**
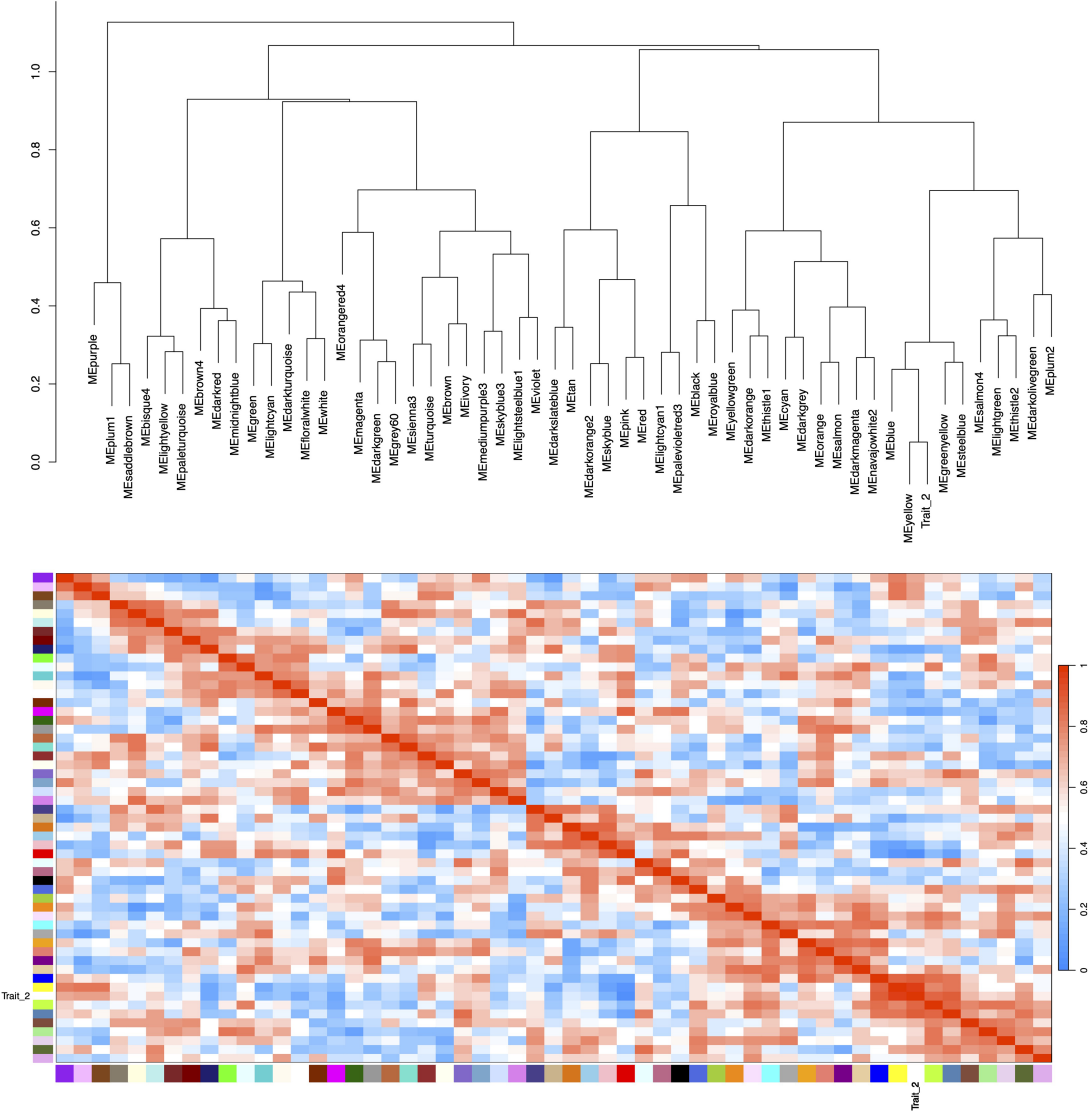
Hierarchical clustering dendrogram and heatmap of module eigengenes. Color scale legend (right of heatmap) displays adjacency values ranging from 0 (blue) to 1.0 (red), with color gradient representing correlation magnitude. Trait_2 corresponds to HB content measured in field-grown plants. Both the dendrogram and heatmap demonstrate a strong association between the yellow module and Trait_2.

Correlation analysis between the 55 merged modules and phenotypic data revealed that the yellow module showed the strongest positive association with the target trait, while the red module exhibited the most significant negative correlation (**Figure 6**). Hierarchical clustering of the merged modules further divided the 55 clusters into six major groups. Notably, the yellow module co-clustered with Trait_2 (**Figure 7**), indicating convergent expression patterns between yellow module genes and Trait_2 phenotypic variation. Based on these findings, we identified the yellow module as key candidate module associated with HB accumulation.

### 3.5 Identification and validation of trait-associated key genes in candidate module

**Figure 8:**
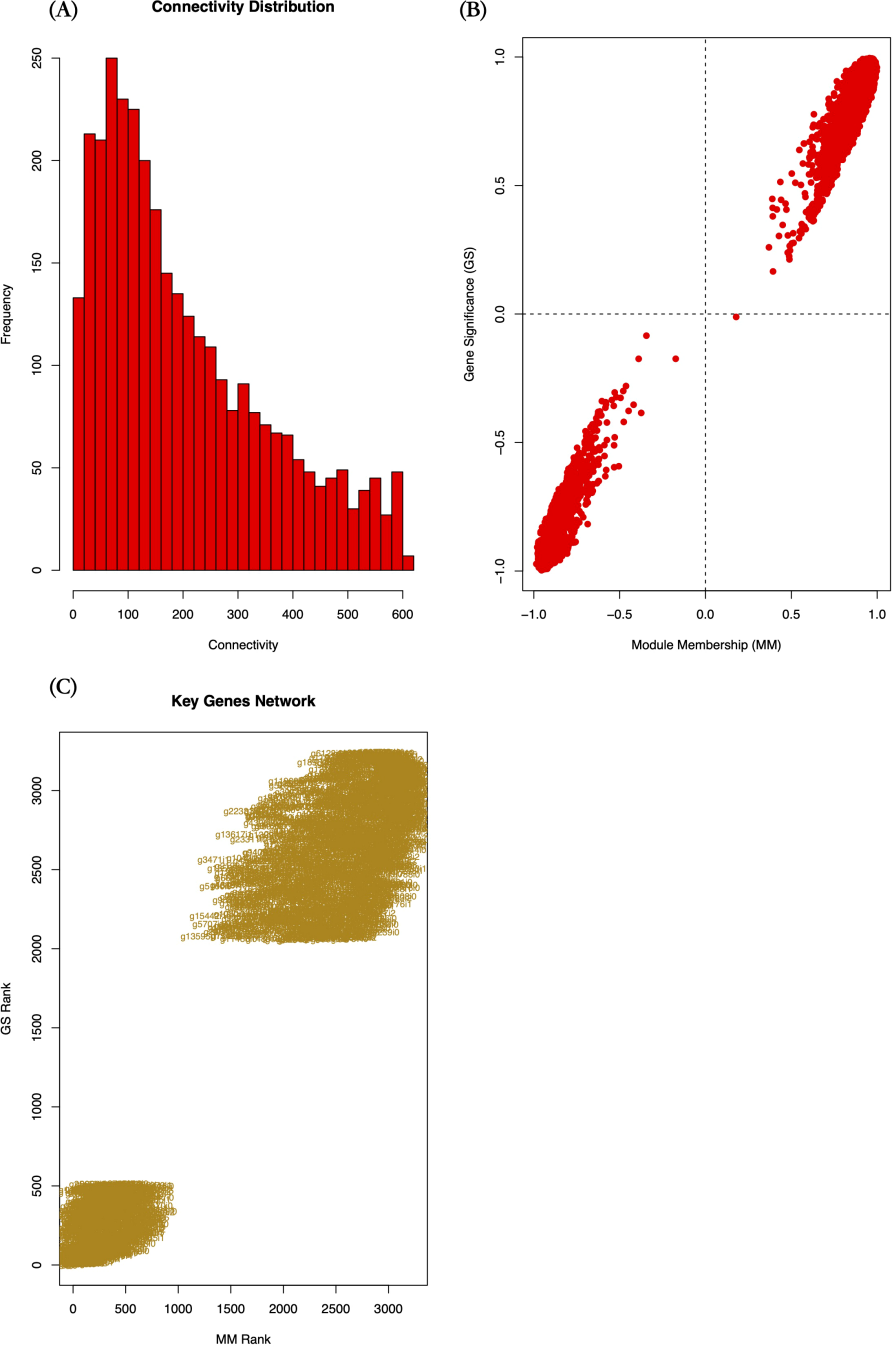
Co-expression network features of candidate module genes. **(A)** Histogram of intramodular connectivity distribution, **(B)** Scatter plot of Gene Significance (GS) vs. Module Membership (MM) values, **(C)** Rank-ordered scatter plot of GS and MM values for key candidate genes. GS measures the association between gene expression and the target phenotype; MM indicates the correlation between genes and module eigengenes; Connectivity reflects the interaction strength of a gene with other module members.

From the candidate yellow module, we extracted 3,240 module genes. To pinpoint key genes strongly associated with the target trait, we performed Gene Significance (GS) and Module Membership (MM) analyses. Analysis revealed that intramodular connectivity of genes ranged from 1.43 to 604.13, with the majority (59.17%) exhibiting connectivity below 200 (**Figure 8A**). Most module genes showed |GS| and |MM| values > 0.5 (**Figure 8B**), indicating both conserved co-expression patterns with the module eigengene, and strong associations with the target phenotype.

Given the large number of genes in the module, we applied stringent selection criteria (|GS| > 0.9, MM > 0.9, p-value of GS < 0.00001) to identify high-confidence candidate genes. This screening yielded 278 key candidate genes (**Supplementary Table S4**), including 60 genes showing inverse correlation with module expression trends (downregulated with increasing HB content), 218 genes exhibiting positive correlation (upregulated with HB accumulation).

**Figure 9:**
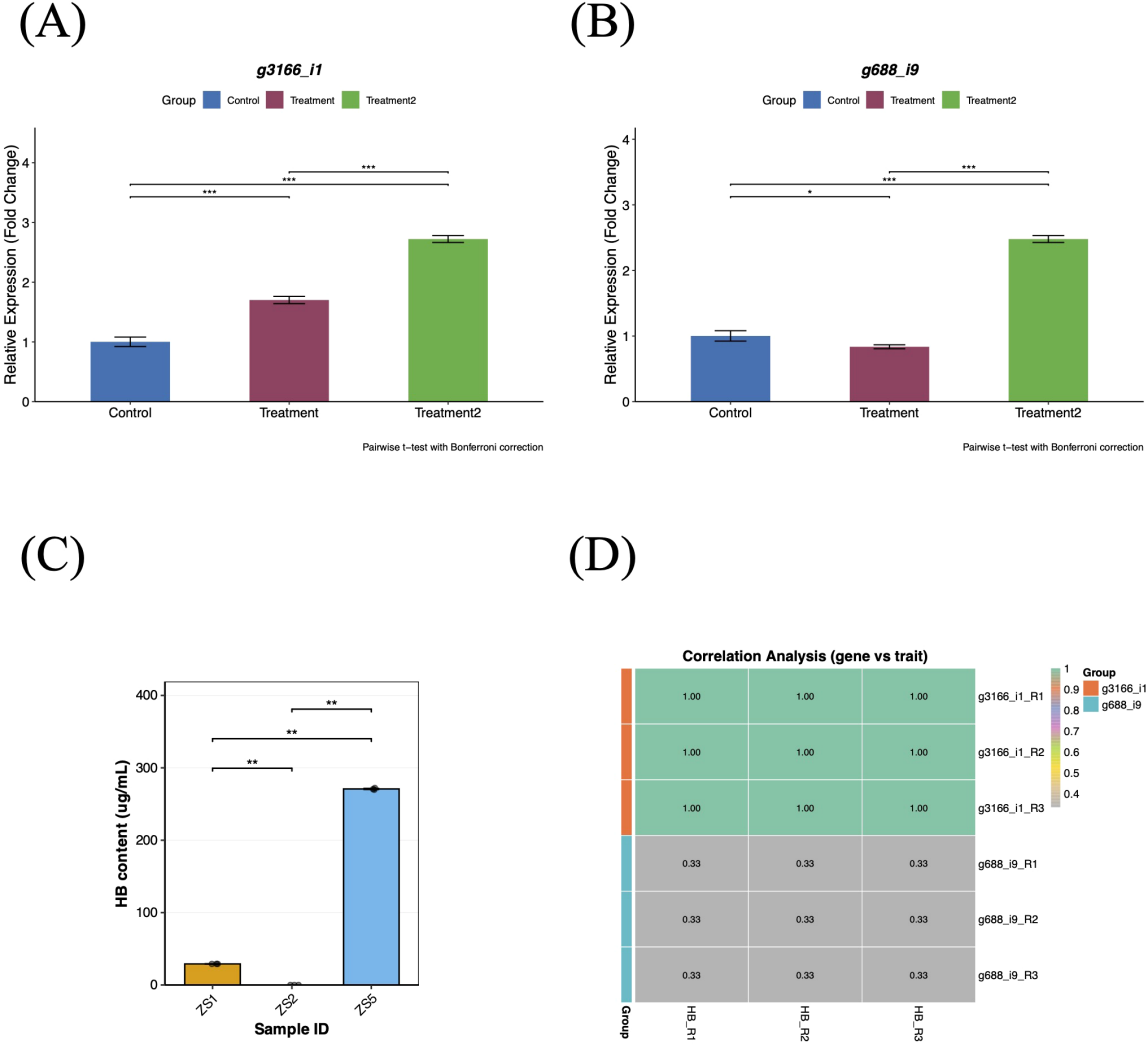
qRT-PCR validation and correlation analysis of candidate genes. **(A)** Relative expression levels of candidate gene *g3166_i1*. **(B)** Relative expression levels of candidate gene *g688_i9*. **(C)** HB content variation among three tissue-cultured samples. **(D)** Heatmap of correlation analysis between candidate genes (*g3166_i1/g688_i9*) and target traits. Control group: ZS2 (HB-deficient); Treatment1: ZS1 (moderate HB); Treatment2: ZS5 (high HB). Expression levels were normalized to *PhACTIN1* using the 2^−ΔΔCt^ method.

To validate the reliability of the transcriptome data, we randomly selected three genes from the top 10% (GS *p-value*) most significant candidate genes (**Table 1**) for qRT-PCR verification using tuberous root tissues of tissue-cultured plants. Candidate gene *g3166_i1* showed significant upregulation in both treatment groups (p < 0.001), with significantly higher expression in ZS5 than in ZS1 (p < 0.001), consistent with the observed HB accumulation patterns (**Figure 9A**). In contrast, candidate gene *g688_i9* exhibited downregulation in ZS1 compared to the control group (p < 0.05) but upregulation in ZS5 (p < 0.001) (**Figure 9B**). Since candidate gene *g14986_i0* was undetectable by qRT-PCR, we performed correlation analysis (Kendall) between the relative expression levels of genes *g3166_i1*/*g688_i9* and HB content. Gene *g3166_i1* displayed a significant positive correlation with HB accumulation (**Figure 9D**), identifying it as a key regulator of HB biosynthesis in *P. heterophylla*.

### 3.6 Sequence characterization and functional annotation of gene *g3166_i1*

**Figure 10:**
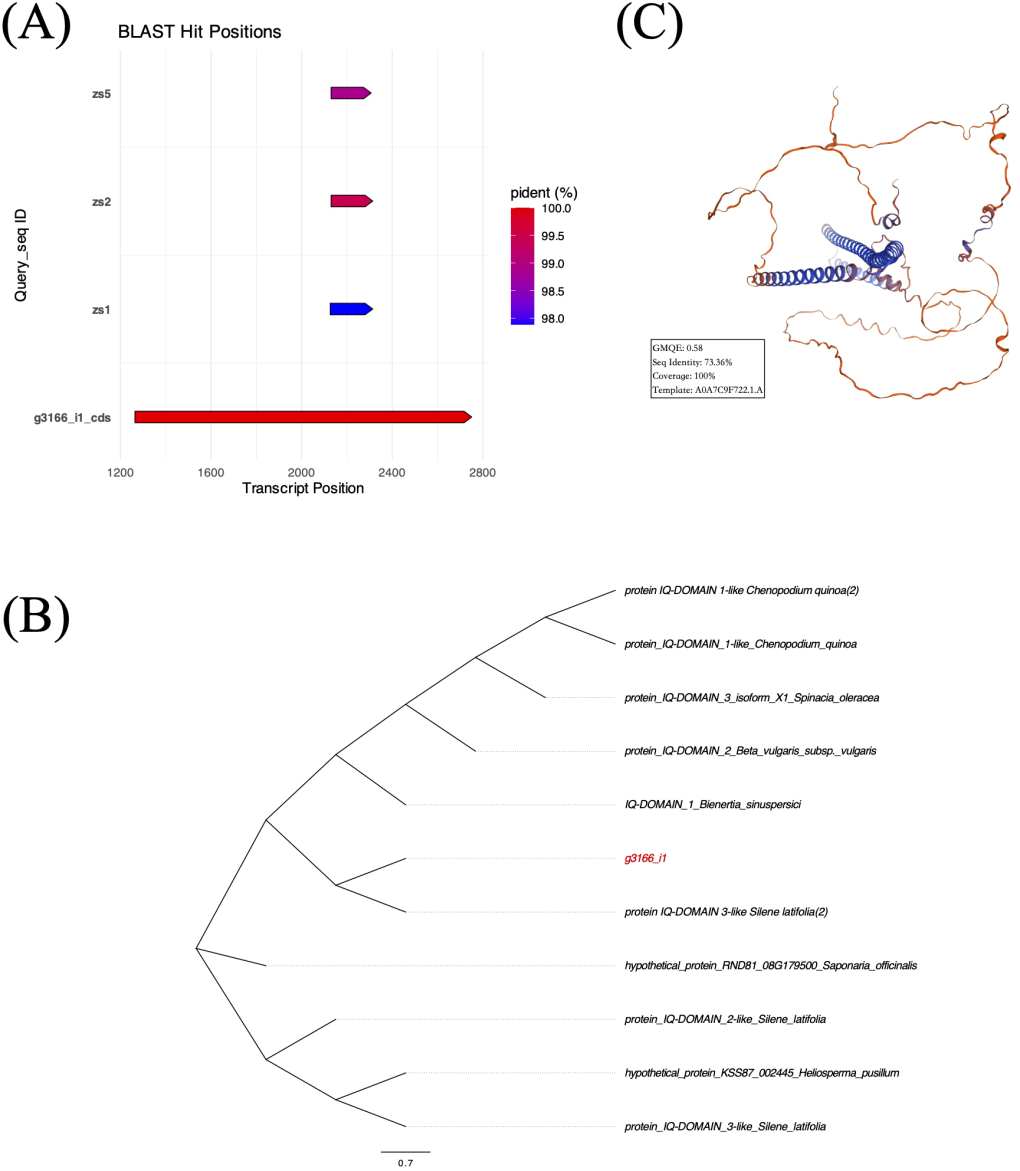
**(A)** Prediction and validation of CDS localization within the transcript of *g3166_i1*. **(B)** Maximum likelihood phylogenetic tree of *g3166_i1* based on top 10 BLASTP hits. **(C)** Predicted tertiary structure of *g3166_i1*-encoded protein by SWISS-MODEL database.

We extracted a 3,068 bp transcript sequence of *g3166_i1* from the reference assembly. Open reading frame (ORF) prediction identified one coding sequence (CDS) spanning transcript 1,264 – 2,751 bp (1,488 bp). Sanger sequencing validation detected this CDS fragment in all three samples with 97.88% – 99.50% identification rates (**Figure 10A**), confirming its biological validity. The CDS of Gene *g3166_i1* encoded a 495-amino acid protein. BLASTP analysis revealed the presence of an IQ domain (**Figure 10B**), consistent with functional annotations from Swiss-Prot, GO, and Pfam databases, suggesting its potential role in calcium ion signal transduction. Tertiary structure prediction using SWISS-MODEL showed 73.36% similarity to the reference protein A0A7C9F722.1.A with 0.58 GMQE value (**Figure 10C**).

## 4 Discussion

### 4.1 Variation in HB accumulation among three types of *P. heterophylla*

Although wild resources of *P. heterophylla* are limited in China, cultivated populations are widely distributed across multiple provinces such as Fujian, Jiangsu, and Guizhou (Li et al., 2024). Significant variations in HB content exist among populations from different geographical origins (Sha et al., 2023). In this study, quantitative HPLC analysis of samples demonstrated undetectable HB levels in ZS2, consistent with previous reports (Zheng et al., 2019), thereby establishing its suitability as a blank control. Furthermore, considering the potential influence of environmental factors on heterophyllin B (HB) accumulation, we performed comparative HPLC analysis of field-grown and tissue-cultured materials. The results demonstrated a consistent reduction in total HB content in tissue-cultured samples (**Figure 9C**) compared to field-grown plants (**Figure 1B**), indicating that field conditions are more conducive to HB biosynthesis. However, the identical hierarchical pattern of HB accumulation across samples (ZS5 > ZS1 > ZS2) under both growth conditions suggests that the fundamental regulatory mechanism of HB variation is genotype-dependent and remains stable in field-cultured and tissue-cultured conditions. This consistency provides a rationale for utilizing tissue-cultured systems in subsequent investigations of HB accumulation mechanisms. Collectively, these three samples represent an ideal material for identifying HB-associated genes: ZS1 with moderate levels, ZS2 with undetectable HB, and ZS5 with high accumulation.

### 4.2 Transcriptome assembly strategy of *P. heterophylla*

The genomic foundation of *P. heterophylla* remains underdeveloped, as no reference genome for this species or the same genus plant is currently available in public databases. Consequently, most previous studies have adopted a *de novo* transcriptome assembly strategy (Li et al., 2016; Qin et al., 2017; Luo et al., 2016) to overcome the absence of reference genomic resources. Fortunately, the chloroplast genome sequence of *P. heterophylla* has been previously reported and is accessible in the GenBank database (Zhang et al., 2023). Given the high conservation of chloroplast genes within plant genera (Bu et al., 2021; Zhang and Feng, 2025), these sequences are typically useful for the identification of closely related or morphologically similar plants (Yan et al., 2023; Chen et al., 2023). In our study, we aligned the *de novo* assembled transcriptome sequences with the retrieved chloroplast genome. Under optimal assembly conditions, the majority or all chloroplast genes should be detectable, serving as a robust indicator of assembly completeness and accuracy. Finally, 73 chloroplast genes (94.81%) were detected in the assembled sequences. Given that the sequencing material consisted of plant tuberous roots where certain chloroplast genes may exhibit low expression or remain transcriptionally inactive. Overall, the alignment result indicates a high quality of the sequence assembly.

### 4.3 Functions implications of gene *g3166_i1* in HB accumulation

Although HB represents a key bioactive compound in *P. heterophylla*, the complete metabolic pathway for its biosynthesis remains elusive. Current transcriptomic studies have identified several genes, including the precursor peptide (*prePhHB*) (Zheng et al., 2019), peptide cyclase (*PhPCY3*) (Qin et al., 2025), and prolyl oligopeptidase (*PhPOP1*) (Shu et al., 2025), that influence endogenous HB accumulation. In our study, the candidate gene (*g3166_i1*) derived from the yellow module encodes a protein annotated across multiple public databases as containing an IQ domain. In plants, five protein families (the IQD family, myosins, CAMTAs, CNGCs, and the IQ motif-containing family) possessing the calmodulin-binding IQ motif have been identified (Zhou et al., 2010). These proteins typically interact with calmodulin (CaM) in a Ca^2+^-independent manner, thereby participating in diverse biological processes including plant development, metabolic regulation, stress responses, and defense mechanisms (Fan et al., 2021; DeFalco et al., 2009). For instance, in *Arabidopsis thaliana*, *AtIQM1* (IQ motif-containing protein 1) indirectly enhances the activity of jasmonic acid (JA) biosynthetic enzymes (ACX2 and ACX3) by promoting the expression of catalase 2 (*CAT2*), consequently increasing JA accumulation (Lv et al., 2019). Interestingly, exogenous application of methyl jasmonate (MeJA) has been confirmed to significantly enhance the expression of *prePhHB*, consequently promoting HB accumulation in *P. heterophylla* (Wu et al., 2024). We therefore propose that the candidate gene *g3166_i1* may enhance HB biosynthesis and accumulation by upregulating *prePhHB* expression through modulation of the JA signaling pathway. Herein, we confirm that *g3166_i1* exhibits significantly upregulation in both ZS1 and ZS5 compared to the control group (**Figure 9A**, p < 0.001). Moreover, its expression levels show a strong positive correlation with HB accumulation across all three samples (**Figure 9D**, τ = 1), suggesting a potential functional role in HB biosynthesis mechanisms. Our findings propose a mechanistic model in which *IQM* genes indirectly regulate HB accumulation in *P. heterophylla* by modulating the JA biosynthetic pathway, thereby revealing a previously unrecognized role of this gene family in HB metabolism within this species.

## 5 Conclusion

In this study, we performed high-throughput sequencing of field-grown *P. heterophylla* samples with contrasting HB content, generating a reference transcriptome comprising 38,288 protein-coding genes. Through WGCNA, we constructed a co-expression network containing 55 distinct modules and identified one key module (3,240 genes) significantly associated with HB accumulation. From this module, 278 key candidate genes were prioritized for further analysis. Experimental validation via qRT-PCR confirmed that the expression pattern of Gene *g3166_i1*, encoding an IQ domain-containing protein, strongly correlated with HB accumulation levels across different samples. These results confirm the potential role of Gene *g3166_i1* in regulating HB biosynthesis in *P. heterophyllla*. Collectively, our findings enable systematic identification of HB biosynthesis-related genes and support molecular breeding strategies for high-HB *P. heterophylla* cultivars.

## Data Availability

The original contributions presented in the study are publicly available. This data can be found here: NCBI, PRJNA1311177.

## Supplementary material

Supplementary Table 1 Alignment results of *P. heterophylla* chloroplast genes to transcript sequences.

Supplementary Table 2 Distribution of different repeat motif SSRs identified in *P. heterophylla* transcriptome.

Supplementary Table 3 Distribution of SNP variants identified in *P. heterophylla* transcriptome.

Supplementary Table 4 Network parameters of key candidate genes in the target module. GS measures the association between gene expression and the target phenotype; MM indicates the correlation between genes and module eigengenes; Connectivity reflects the interaction strength of a gene with other module members.

Supplementary Table 5 The sample information of three *P. heterophylla*.
